# WHODAS 2.0 as a Measure of Severity of Illness: Results of a FLDA Analysis

**DOI:** 10.1155/2018/7353624

**Published:** 2018-03-25

**Authors:** Alba Sedano-Capdevila, María Luisa Barrigón, David Delgado-Gomez, Igor Barahona, Fuensanta Aroca, Inmaculada Peñuelas-Calvo, Carolina Miguelez-Fernandez, Alba Rodríguez-Jover, Susana Amodeo-Escribano, Marta González-Granado, Enrique Baca-García

**Affiliations:** ^1^Department of Psychiatry, IIS-Jiménez Díaz Foundation, Madrid, Spain; ^2^Department of Psychiatry, Autónoma University, Madrid, Spain; ^3^Departamento de Estadística, Universidad Carlos III, Getafe, Madrid, Spain; ^4^Instituto de Matemáticas, Universidad Nacional Autónoma de México, Ciudad de México, Mexico; ^5^Department of Psychiatry, University Hospital Rey Juan Carlos, Móstoles, Spain; ^6^Department of Psychiatry, General Hospital of Villalba, Madrid, Spain; ^7^Department of Psychiatry, University Hospital Infanta Elena, Valdemoro, Spain; ^8^CIBERSAM (Centro de Investigación en Salud Mental), Carlos III Institute of Health, Madrid, Spain; ^9^Universidad Católica del Maule, Talca, Chile

## Abstract

WHODAS 2.0 is the standard measure of disability promoted by World Health Organization whereas Clinical Global Impression (CGI) is a widely used scale for determining severity of mental illness. Although a close relationship between these two scales would be expected, there are no relevant studies on the topic. In this study, we explore if WHODAS 2.0 can be used for identifying severity of illness measured by CGI using the Fisher Linear Discriminant Analysis (FLDA) and for identifying which individual items of WHODAS 2.0 best predict CGI scores given by clinicians. One hundred and twenty-two patients were assessed with WHODAS 2.0 and CGI during three months in outpatient mental health facilities of four hospitals of Madrid, Spain. Compared with the traditional correction of WHODAS 2.0, FLDA improves accuracy in near 15%, and so, with FLDA WHODAS 2.0 classifying correctly 59.0% of the patients. Furthermore, FLDA identifies item 6.6 (illness effect on personal finances) and item 4.5 (damaged sexual life) as the most important items for clinicians to score the severity of illness.

## 1. Introduction

Having accurate indicators that measure the impact of illnesses on people's live is a critical issue in several areas of medicine, including mental health. Disability is a useful construct for this. Disability refers to the difficulty of people suffering a disease to keep their premorbid or normal functionality. The World Health Organization (WHO) describes disability as a difficulty in functioning at the body, person, or societal levels, in one or more life domains, as experienced by an individual with a health condition in interaction with contextual factors [1]. To know the degree of disability helps clinicians to measure the impact of being ill for a specific patient, to decide in which areas a person needs help and to evaluate treatment effectiveness.

The need to quantify disability first appears in 1962, with the publication of Health-Sickness Rating Scale (HSRS) [2]. This scale was replaced by the Global Assessment Scale (GAS) in 1976 [3] which was further reviewed as the Global Assessment of Functioning Scale (GAF), included in the DSM-III and DSM-IV [4]. GAF is a scale which is still frequently used to measure a person's psychological, social, and occupational functioning on a hypothetical continuum of mental health-illness ranging from 1 to 100; simplicity and unidimensionality of GAF have been proposed as a strength of this scale [5]. In DSM-IV is also included Social and Occupational Functioning Assessment Scale (SOFAS) as a functionality measure, but an important weakness of this scale is that it does not consider symptoms severity [5].

In response to the need to have a tool to evaluate functionality with a cross-cultural perspective and at the same time be easy to apply, WHO developed the World Health Organization Disability Assessment Schedule (WHODAS), and its next version, with more domains, WHODAS 2.0 [6]. Currently, DSM-5 recommends the replacement of GAF by WHODAS 2.0 in order to increase the reliability of disability scores. WHODAS 2.0 has high internal consistency, high test-retest reliability, and good concurrent validity in patient classification when compared with other recognized disability measurement instruments. Nevertheless, WHODAS has certain limitations. It is not valid for children and youth and bodily impairments and environmental factors are not measured [7]. WHODAS has been translated into more than ten languages; it is useful in the evaluation of disability in mental health conditions but also in a wide range of physical health diseases [8]. The demonstrated reliability during its use favored its inclusion in DSM-5.

In routine clinical practice, clinicians generally classify patients' illness severity according to their clinical experience and are supported by severity criteria used in measurement scales and classification manuals. Due to time restrictions in clinical practice, use of scales and questionnaires is limited. Simple scales such as the Global Clinical Impression Scale (CGI) allow the clinician to measure the severity and evolution of a patient without too much impact on the clinician's care and clinical activity. CGI is an evaluation method for seriousness of symptoms in mental illnesses. The scale is composed by three global measures: severity of illness at the moment of evaluation (CGI-S); global improvement since last visit (CGI-I), and an efficacy index useful to compare the premorbid status and severity of treatment side effects (CGI-E). It is commonly used in clinical trials in depression or schizophrenia [9, 10] or to be compared with other instruments like, for example, Beck Depression Inventory [11]. Nonetheless, CGI validity has been questioned and CGI is occasionally pointed as an inconsistent, unreliable, and too general measure [12–14].

Although the relationship between illness severity and functionality or disability has been widely studied in mental disorders such as schizophrenia [15], studies using these two particular questionnaires, WHODAS 2.0 and ICG, are scarce and all previous works have used standard statistical techniques. Using WHODAS 2.0, Bastiaens et al. demonstrated a significant correlation between CGI and WHODAS 2.0 in patients with dual disorders [16] and Guilera et al. found a positive correlation between CGI and WHODAS 2.0 subscales [17].

In the present study, we use Fisher Linear Discriminant Analysis (FLDA), a pattern recognition method [18] to explore if WHODAS 2.0 can be used for identifying severity of illness measured by CGI-S in a sample of outpatients in mental health facilities evaluated in real clinical practice and for identifying which individual items of WHODAS 2.0 are more discriminant for severity of illness classification. Furthermore, we hypothesized that FLDA would improve the accuracy of WHODAS 2.0.

## 2. Materials and Methods

### 2.1. Setting and Participants

From January to March 2017, a sample of 122 patients was evaluated in routine psychiatric or psychological visits at mental health facilities affiliated with the Fundación Jiménez Díaz Hospital in Madrid, Spain (Rey Juan Carlos Móstoles Hospital, Infanta Elena Valdemoro Hospital, General Hospital of Villalba, and University Hospital Fundación Jiménez Díaz).

All patients attended in the Psychiatry Department were candidates to participate in the study as long as they met the following inclusion criteria: outpatients, aged 18 or older, and who gave written informed consent. Exclusion criteria were illiteracy, refusal to participate, and situations in which the patient's state of health did not allow for written informed consent.

All clinicians (psychiatrists, psychologists, and mental health nurses) were trained in the use of WHODAS 2.0 and ICG in December 2016 in a consensus meeting and after that, all of them were encouraged to use the instruments in their daily clinical practice. They were all asked to assess between 5 to 7 patients. Thirty-one clinicians participated actively in patient's recruitment and they included a mean of 5.5 ± 4.3 patients.

### 2.2. Assessment

CGI and WHODAS 2.0 were used to assess all patients, in an electronical version integrated in MEmind (https://www.memind.net), a web-based platform used in the Psychiatry Department since May 2014 as part of the standard clinical activity [19]. At the end of 2016, all clinicians were trained in the use of WHODAS 2.0 and were instructed to use it in addition to usual questionnaires in a free way. In this way, until the end of March 2017, 122 patients were randomly selected and assessed.

WHODAS 2.0 [8] arises after recognizing the difficulty in the daily clinical practice to use ICF; it is translated to more than ten languages, including Spanish [20]. Symptoms of disability are divided into six domains with several items in each one. For every item, users have to answer how much difficulty they have had in the last 30 days to do something. Items are scored from one to five: 1 (none difficulty), 2 (mild), 3 (moderate), 4 (severe), and 5 (extremely difficult/cannot). WHODAS 2.0 is composed by 36 items: 6 in the “cognition domain,” 5 in “mobility domain,” 4 items in “self-care,” 5 questions on “getting alone and the interaction with the others,” 8 items about “life activities,” and last domain with 8 questions about “joining in community activities.” In this study, we used the 36-item interviewer-administered version of WHODAS 2.0, which scores from 0 to 100 with higher scores reflecting greater disability.

CGI is an instrument to assess the severity of symptoms of mental disease according to the judgment of the clinician [21, 22]. CGI is composed of three measures: CGI-S, CGI-I, and CGI-E. With CGI-S, the measure employed in this study, the observer describes the severity of illness at the present moment in a 7-point Likert scale from 1 (normal, nonillness) to 7 (most gravity of disease). We divided score in three groups of severity: 1 to 4 representing low severity; 4 representing medium severity; and 6-7 as the worst group according to severity.

Furthermore, information on sociodemographics and ICD 10 diagnosis was collected.

### 2.3. Ethical Issues

This study was conducted in compliance with the Declaration of Helsinki and approved by the IRB at Fundación Jiménez Díaz Hospital. All patients who participated in the study signed an informed consent that was detailed by the clinician who did the assessment.

Concerning data protection, access to the online user interface was restricted to participating clinicians (MEmind Study Group). The data provided by the clinician was encrypted by Secure Socket Layer/Transport Layer Security (SSL/TLS) between the investigator's computer and the server. Data was stored in an external server created for research purposes. An external auditor guaranteed that security measures met the Organic Law for Data Protection standards at a high protection level.

### 2.4. Statistical Analysis

In the pattern recognition community, Fisher Linear Discriminant Analysis (FLDA) [18] is one of the most used analytical tools to transform the raw data into a lower dimensional subspace by maximizing a class separation criterion. Concisely, if the data contain *n* observations belonging to *m* possible classes, this technique finds *L* linear projections (*L* = min⁡(*n*, *m*)) in such a way that the class separation is maximized and the intraclass variation minimized. Before applying the FLDA algorithm, a principal component analysis keeping 95% of the variance was applied to remove noise [23]. Blasco-Fontecilla et al. [24] used this technique to readjust the Holmes and Rahe stress inventory to successfully discriminate controls from suicide attempters.

Once the data has been transformed into a more suitable space, we use the* k*-nearest neighbour classifier to determine the class of a new observation. This classifier finds the *k* observations with less distance to the new observation and assigns the majority class of these *k* observations to the new one. In this article, the Euclidean distance is used and we consider *k* is equal to 1, 3, 5, and 7.

A *K*-fold cross-validation set-up was carried out to evaluate the classification accuracy of this approach (FLDA + *k*-nearest neighbour). In this article, we use *K* = *n*. That is, *n* − 1 observations were used to conduct the FLDA and the *k*-nearest neighbour and the holdout observation was used to test the performance of the classifier. This process was repeated *n* times, once for each observation that is left out.

## 3. Results and Discussion

### 3.1. Sample Description

The sample contains 55 (45.1%) men and 67 (54.9%) women, with a mean age of 49 ± 17.5 years. Concerning civil status, 63 patients (51.6%) were married whereas the rest were single, divorced, or widower. Concerning occupation, 70 patients (59.3) were active population.

Table 1 shows ICD 10 diagnosis of patients. There were 171 different diagnoses as some patients had comorbid diagnosis.  Table 2 shows the scores for CGI.

When we performed Pearson test for study correlation, we found a low positive correlation between CGI-S and total WHODAS 2.0 (*r* = 0.16; *p* = 0.06). This result contrasts with results of previous studies, which have found higher correlations: 0.48 in the study on 100 patients with dual diagnoses in a community correctional treatment [16] and correlation indexes between CGI and the different domains of WHODAS ranging from 0.341 (self-care) to 0.629 (participation) in 291 patients with bipolar disorder [17]. As it is explained later, this lower correlation might be explained by the fact that we analyzed a more general population than these previous works.

### 3.2. FLDA Analyses

**Figure 1:**
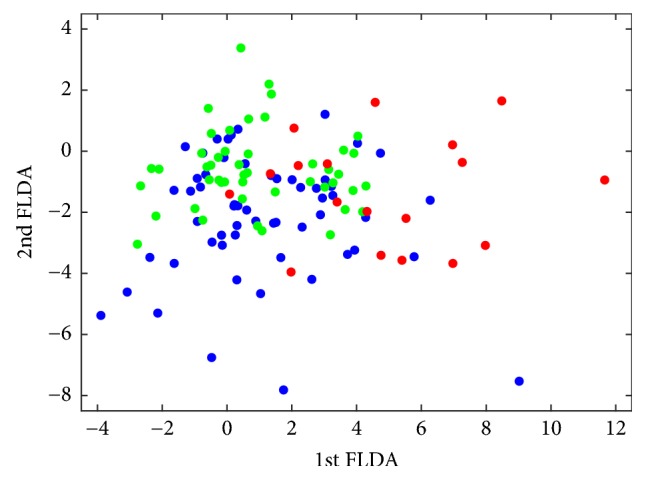
Scatter plot of the FLDA scores. Green dots represent ICG-S from 1 to 4 (low severity). Blue dots represent ICG-S of 5 (medium severity). Red dots represent ICG-S of 6 or 7 (high severity).

We performed a Fisher Linear Discriminant Analysis and obtained the weights of individual items for each projection (Table 3) and the scattered plot for FLDA scores (Figure 1).

In Table 3 and Figure 1, we can observe that higher scores in the first projection imply more illness severity, represented with red dots. That means that individual items with higher positive values are the most important when clinicians assign patients a worse clinical conditions. Specifically, the two items related to a high level of severity of illness were item 6.6 (weight = 1.3728) and item 4.5 (weight = 0.6378), which means that patient in whom illness has a negative effect on personal finances (item 6.6) or has damaged sexual life (item 4.5) tends to be scored as severely ill or among the most extremely ill patients by their doctors. Additionally, in the figure can be recognized differentiated groups but also areas of overlapping are clear. This is not surprising as ICG-S has been pointed out to have some limitations [12–14], and some authors have found ICG does not correlate well with other measures of severity of illness in depression [14] or dementia [13].

In order to determine the accuracy attained by our FLDA/*k*-nearest neighbour approach and to discover if this approach improves the accuracy obtained by the standard clinical approach, we performed a cross-validation experiment.  Table 4 shows the classification accuracy of both FLDA and clinical approach in a 122-fold cross-validation experiment. In this table, we can notice that FLDA obtains a better accuracy than the clinical approach (score WHODAS 2.0 in the traditional way) for any *k* considered. In addition, the best value is obtained when we use 3 neighbours.

**Figure 2:**
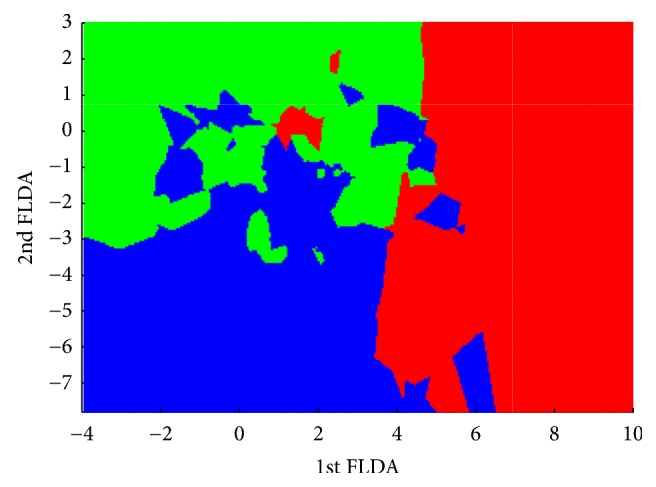
Classification map.

Finally, we make a classification map for the best result (*K* = 3) which is showed in Figure 2. In this map, we observe the existence of some “islands” as a consequence of the previously described overlapping.

## 4. Conclusion

We found that WHODAS 2.0 is a useful scale for measuring severity of illness scored by clinicians with ICG, and so WHODAS 2.0 correctly classifies 59.0% of the patients. Compared with the traditional correction of WHODAS 2.0, FLDA improves accuracy in near 15% with respect to the traditional method. However, as it is shown in the classification map figure, the classification is far from being perfect and there are overlapped areas and some patients can be catalogued by WHODAS 2.0 with a low level of illness severity whereas clinicians classified them with higher scores and vice versa. Finally, FLDA shows that there are certain items of WHODAS more important for clinicians when considering severity of illness, specifically items regarding economic repercussion of illness and regarding a detriment of sexual life.

In contrast with previous studies, our sample is composed of patients obtained in a real clinical environment with a range variety of diagnoses which represent one strength of our study. To develop studies in real clinical settings is important as this gives us a useful insight for a daily practice. Furthermore, we do not just study correlations between CGI and WHODAS 2.0 but use a more sophisticated statistical method and demonstrated that FLDA is useful for better classification of illness severity of patients using a disability measure, in a similar way that we previously did in the field of suicide [24]. Consequently, we proposed this statistical method as a promising method to be used in the field of mental health and in other areas of health.

However, our study also has certain limitations. First, our sample size was relatively small, which in part is influenced by data collection method as MEmind web platform is time consuming for a clinician. Moreover, while the range variety of diagnoses composing our sample is a strength, this heterogeneity can also be considered a limitation. As the impact on the disease in the functionality is very different in every mental disorder, a further analysis differentiating by diagnosis would be necessary, but unfortunately our sample size does not allow us to do that. This point should be taken into account as a future perspective of our work.

In conclusion, in this study we demonstrated an association between WHODAS 2.0 and ICG in a group of patients heterogeneously diagnosed. Future works focusing on this relationship in particular diagnoses are warranted.

## Figures and Tables

**Table 1:** Diagnoses of total sample.

Mental and behavioural diagnoses	*N*	Percent
Schizophrenia	24	14
Delusional disorder	7	4.09
Unspecified nonorganic psychosis	4	2.33
Schizoaffective disorders	5	2.92
Schizotypal disorder	1	0.58

Bipolar affective disorder	12	7.01
Depressive episode	8	4.67
Dysthymia	8	4.67

Adjustment disorders	10	5.84
Mixed anxiety and depressive disorder	13	7.60
Panic disorder	1	0.58
Specific (isolated) phobias	2	1.16
Agoraphobia	1	0.58
Dissociative disorders	1	0.58
Obsessive-compulsive disorder	1	0.58
Hypochondriacal disorder	1	0.58
Posttraumatic stress disorder	1	0.58
Somatoform disorders	2	1.16
Neurasthenia	1	0.58

Mental and behavioural disorders due to use of alcohol	9	5.26
Mental and behavioural disorders due to use of cannabinoids	7	4.09
Mental and behavioural disorders due to use of cocaine	3	1.75
Mental and behavioural disorders due to use of opioid	1	0.58
Mental and behavioural disorders due to use of sedatives or hypnotics	1	0.58
Pathological gambling	1	0.58

Personality disorder	15	8.77
Anorexia nervosa	2	1.16
Disturbance of activity and attention	8	4.67
Mild mental retardation	1	0.58
Sexual dysfunction, not caused by organic disorder or disease	2	1.16

Other diseases	*N*	Percent

Essential (primary) hypertension	3	1.75
Human immunodeficiency virus [HIV] disease	2	1.16
Malignant neoplasm of breast	2	1.16
Angina pectoris	1	0.58
Diabetes Mellitus	1	0.58
Generalized pain	1	0.58
Hearing loss, unspecified	1	0.58
Hypothyroidism	2	1.16
Thalassaemia	1	0.58
Chronic hepatitis	1	0.58
Diabetes polyneuropathy	1	0.58
Chronic prostatitis	1	0.58
Dizziness	1	0.58

**Table 2:** ICG-S measured by the clinician.

Score	*N*	Percentage
Normal, not at all ill (1)	5	4.10
Borderline mentally ill (2)	3	2.46
Mildly ill (3)	4	3.28
Moderately ill (4)	35	28.69
Markedly ill (5)	57	46.72
Severely ill (6)	14	11.48
Among the most extremely ill patients (7)	4	3.28

**Table 3:** Weights assigned by FLDA algorithm to individual items in the two projections.

Domain	Items: in the last 30 days, how much difficulty did you have in:	Weight for 1st FLDA	Weight for 2nd FLDA
(1) Cognition	(1.1) Concentrating on doing something for 10 minutes	−0.2434	0.1333
	(1.2) Remembering to do important things	−0.2597	0.2349
	(1.3) Analysing and finding solutions to problems in day to day life	−0.0663	0.2974
	(1.4) Learning a new task, for example, learning how to get to a new place	0.4333	−0.7471
	(1.5) Generally understanding what people say	0.2884	0.0369
	(1.6) Starting and maintaining a conversation	−0.1467	0.3423

(2) Mobility	(2.1) Standing for long periods such as 30 minutes	−0.4067	−0.0713
	(2.2) Standing up from sitting down	0.2553	0.1258
	(2.3) Moving around inside your home	0.0595	0.0485
	(2.4) Getting out of your home	0.2897	0.0663
	(2.5) Walking a long distance such as a kilometre	0.1169	−0.3475

(3) Self-care	(3.1) Washing your whole body	−0.4082	−0.1384
	(3.2) Getting dressed	−0.3430	−0.0682
	(3.3) Eating	−0.4251	−0.0252
	(3.4) Staying by yourself for a few days	0.2476	−0.0575

(4) Getting along	(4.1) Dealing with people you do not know	−0.0020	0.4178
	(4.2) Maintaining a friendship	−0.0066	−0.5309
	(4.3 Getting along with people who are close to you	−0.0756	−0.4095
	(4.4) Making new friends	−0.4115	−0.1626
	(4.5) Sexual activities	0.6378	−0.0650

(5) Life activities	(5.1) Taking care of your household responsibilities	−0.0822	−0.3258
	(5.2) Doing most important household tasks well	0.4353	0.0420
	(5.3) Getting all the household work done that you needed to do	0.2727	0.1454
	(5.4) Getting your household work done as quickly as needed	0.1028	0.3301
	(5.5) Your day-to-day work/school	−0.2479	−0.2114
	(5.6) Doing your most important work/school tasks well	−0.1420	0.0146
	(5.7) Getting done all the work that you needed to do	0.0867	−0.1365
	(5.8) Getting your work done as quickly as needed	0.0814	0.2283

(6) Participation	(6.1) Joining in community activities	−0.2835	0.1657
	(6.2) Because of barriers or hindrances in the world	−0.3028	−0.4451
	(6.3) Living with dignity	0.4585	0.4136
	(6.4) From time spent on health condition	−0.3687	0.2776
	(6.5) Feeling emotionally affected	−0.0627	0.1088
	(6.6) Because health is a drain on your financial resources	1.3728	0.1791
	(6.7) With your family facing difficulties due to your health	0.1165	−0.0106
	(6.8) Doing things for relaxation or pleasure by yourself	−0.3320	−0.5772

**Table 4:** Classification accuracy of FLDA and clinical approaches. *k* represents the number of considered nearest neighbours.

*K*	1	3	5	7
FLDA	45.9	59.0	53.3	45.9
Clinical approach	45.9	40.1	42.6	36.9
